# Editorial: Recent advances in mechanisms and therapeutics for Fragile X Syndrome and autism

**DOI:** 10.3389/fnins.2023.1187799

**Published:** 2023-05-12

**Authors:** Gemma Molinaro, Kimberly M. Huber, Elizabeth A. McCullagh, Sophie R. Thomson

**Affiliations:** ^1^Department of Neuroscience, O'Donnell Brain Institute, University of Texas Southwestern Medical Center, Dallas, TX, United States; ^2^Department of Integrative Biology, Oklahoma State University, Stillwater, OK, United States; ^3^Centre for Discovery Brain Sciences, University of Edinburgh, Edinburgh, United Kingdom

**Keywords:** E/I imbalance, autism spectrum disorders (ASDs), Fragile X Syndrome (FXS), therapeutics, microbiome

## Introduction

There are potentially many convergent phenotypes across forms of autism spectrum disorder (ASD) providing insight into common mechanisms that underlie these complex disorders; specifically the use of syndromic or monogenic forms of ASD have been particularly insightful. The most common monogenic form of intellectual disability and ASD is Fragile X Syndrome (FXS). While there has been extensive research into the underlying causes of FXS and a myriad of therapeutic approaches attempted, there is still no cure and there are continued advances being made that will broaden knowledge in FXS and ASD. This special edition highlights signaling pathways involved in FXS, including a continued understanding of excitatory/inhibitory imbalances, new targets for therapeutic strategies including calcium modulation and dietary interventions.

## Cell fate alterations and hyperexcitability in FXS

Motor hyperexcitability and repetitive behaviors are associated with Fragile X Syndrome (FXS) and these phenotypes have been recapitulated in many animal and cell models of FXS. It is hypothesized that an excitation/inhibition (E/I) imbalance gives rise to the observed hyperexcitability, with a reduction in GABAergic signaling often cited as the underlying cause. Though importantly, the one-dimensional E/I model has been recently updated to reflect further dimensionality of shifts in E/I that occur during development and complexity of neuronal circuitry (O'Donnell et al., 2017). As FMRP has been linked to cell fate determination in several FXS models (Yang et al., 2007; Liu et al., 2018; Doll et al., 2021; Raj et al., 2021), Barker et al. hypothesized that loss of Fmrp in zebrafish motor neuron progenitors affects the proportion of inhibitory interneurons generated in the developing spinal cord, leading to an E/I imbalance.

Barker et al. found an increase in GABAergic interneurons and inhibitory synaptic proteins during early embryogenesis in *fmr*1^−/−^ zebrafish embryonic spinal cord, with the excessive production of GABAergic interneurons originating from motor neuron progenitors. These cells were further characterized and determined to be early ventral lateral descending (VeLD) cells—the cell type which acts as central pattern generators early in development. While functional GABA responses were not analyzed in this study, Barker et al. did find reduced expression of proteins associated with inhibitory synapses suggesting that early changes in E/I balance due to loss of Fmrp may result in persistent hyperexcitability of neuronal circuits.

These early findings from Barker et al. give rise to many questions regarding motor unit formation in the absence of Fmrp and the mechanisms underlying E/I imbalance. Importantly, this study provides insight into how early consequences of Fmrp loss may have long lasting or permanent effects. These early developmental studies have important implications for timing interventions and efficacy outcome measures for any potential therapeutic for FXS.

## Chloride imbalance in Fragile X Syndrome

E/I imbalance underlies central nervous system alterations in many brain areas seen in FXS and is often a convergent phenotype with other neurodevelopmental disorders (NDDs) such as autism spectrum disorder (ASD). Another addition from the Doll lab in the form of a review highlights the critical role for chloride (Cl-) transporters in neurodevelopment and as important modulators of E/I balance demonstrating an important potential consideration in NDDs such as FXS and ASD and adding to the multi-dimensionality of E/I balance models in ASD. Cl- transporters and Cl- dynamics shift during development with GABAergic signaling changing from excitatory to inhibitory transmission. In FXS, and other NDDs, this polarity shift of GABA is delayed potentially through Cl- transporter dysregulation (Talos et al., 2012; He et al., 2014; Banerjee et al., 2016; Amin et al., 2017). Depolarizing GABA acts during neurodevelopment to regulate neurogenesis, synaptogenesis, E/I balance, and glutamatergic synapse development, which if delayed or dysfunctional can lead to altered neurotransmission such as in FXS and other NDDs. Further, the review discusses non-canonical roles for the Cl- transporter Kcc2 such as dendritic spine structure and function through actin interactions and postsynaptic modulation through physical interactions with scaffolding proteins of postsynaptic receptors. The importance during neurodevelopment and functional synaptic and morphological roles demonstrate that Cl- transporters may be considered for pharmacological treatment of FXS and other NDDs. However, the complexity of E/I signaling and heterogeneity of NDDs must be considered for pharmacological approaches to be successful.

## Neuronal calcium sensor pathway as a treatment for FXS

Neurodevelopmental disorders such as FXS result in lifelong cognitive and behavioral deficits and represent a major public health burden. Alterations in synaptic function in FXS have been characterized and rescued in animal models using genetic and pharmacological approaches. However, despite great effort in understanding the cell biology of FXS, currently there is no approved or effective treatment that targets the mechanisms underlying FXS.

In their work, Cogram et al. decided to take a different, more general, approach by focusing on rebalancing synaptic dysfunction instead of focusing on correcting FMRP-regulated genes directly. They reasoned that affecting calcium signaling globally may improve synaptic homeostasis imbalance in FXS, especially as calcium signaling is impacted in FXS. In particular, they found that the inhibition of the neuronal calcium sensor, NCS-1/Ric8a, by a small heterocyclic compound, the phenothiazine FD44, has great potential therapeutic benefits for FXS and potentially other neurodevelopmental disorders. FD44 crosses the blood–brain barrier and after a daily intraperitoneal dose of 10 mg/ kg for 4 weeks, can rescue many behavioral phenotypes caused by FMRP losses such as hyperactivity, anxiety, repetitive behavior, aggression, and social dysfunction. Since it has been suggested that dopaminergic regulation may be implicated in modulation of social behavior, the authors also examined dopamine metabolism and found it to be increased in the limbic area of the FXS mouse model. Remarkably, they also found that this neurotransmitter dysregulation can be reversed by FD44 treatment.

Considering the findings of Cogram et al. combining strategies to correct both direct *Fmr1* regulated mechanisms as well as indirect downstream pathways may represent a successful way to improve symptoms and quality life in patients with FXS.

## Effects of diet and the microbiome in ASD and Fragile X

A burgeoning area of research in ASD is the role of the gut-brain axis in the etiology of ASD behaviors and pathophysiology. Alam et al. review the latest findings on the role of diet, including vitamins (micronutrients) and pre- and probiotics, on the development and treatment of ASD behaviors in clinical trials, case studies and in genetic and environmental animal models of autism. While this field is still developing, there are some encouraging findings that warrant follow up to determine the applicability in a broad ASD population and to understand mechanisms. Specifically, Alam et al. discuss the positive results of a ketogenic diet, as well as the less restrictive, gluten-free, casein-free diet, that improves behaviors in individuals with ASD and genetic mouse models, such as the Shank3 model of Phelan-McDermid Syndrome and the *Fmr1* KO model of FXS. They provide insight into the metabolic consequences of these diets in individuals with ASD and potential for blood based biomarkers.

Although there are fewer studies on diet in Fragile X Syndrome (FXS), there is some interesting and surprising new research in this area. Supplementation of *Fmr1* KO models with omega-3 fatty acids, surprisingly, rescues many behavioral phenotypes and inflammatory biomarkers in the brain. Alam et al. also discuss the effects of a soy-protein diet, including soy formula on the developmental onset and exacerbation of ASD behaviors and epilepsy in children and *Fmr1* KO mice. Lastly, Alam et al. discuss the varied results of pre- and probiotics in the ASD individuals and animal models. Generally, probiotics appear to have potential in improving ASD behaviors and gastrointestinal (GI) problems, but much larger and more controlled preclinical and clinical studies are needed. Future directions in nutrition and gut-brain interactions in ASD are suggested that address gaps in this exciting and growing area of research.

## Conclusion

In conclusion, recent advances highlight the continued importance of monogenic syndromes such as FXS in understanding pathways involved in neurodevelopmental conditions, hopefully leading to new therapeutic strategies. In addition, many of the affected pathways may be convergent across forms of ASD, and common treatment such as dietary interventions could be utilized. Future work involving mechanisms and pathways that are convergent between animal models and humans, using a diversity of experiments to target pathways involved, and continued understanding across circuitry (including motor systems), personalized medicine approaches and finally larger clinical trials are needed to continue research in these complex disorders.

## Author contributions

All authors listed have made a substantial, direct, and intellectual contribution to the work and approved it for publication.
